# Low extracellular potassium prolongs repolarization and evokes early afterdepolarization in human induced pluripotent stem cell-derived cardiomyocytes

**DOI:** 10.1242/bio.024216

**Published:** 2017-06-15

**Authors:** Jukka Kuusela, Kim Larsson, Disheet Shah, Chandra Prajapati, Katriina Aalto-Setälä

**Affiliations:** 1 Institute of Biomedical Technology, University of Tampere, Tampere, Finland; 2 BioMediTech, Tampere, Finland; 3 School of Medicine, University of Tampere, Tampere, Finland; 4 Heart Hospital, Tampere University Hospital, Tampere, Finland

**Keywords:** Induced pluripotent stem cells, Long QT syndrome, Hypokalemia, Patch clamp, Multielectrode array

## Abstract

Long QT syndrome (LQTS) is characterized by a prolonged QT-interval on electrocardiogram and by increased risk of sudden death. One of the most common and potentially life-threatening electrolyte disturbances is hypokalemia, characterized by low concentrations of K^+^. Using a multielectrode array platform and current clamp technique, we investigated the effect of low extracellular K^+^ concentration ([K^+^]_Ex_) on the electrophysiological properties of hiPSC-derived cardiomyocytes (CMs) generated from a healthy control subject (WT) and from two symptomatic patients with type 1 of LQTS carrying G589D (LQT1A) or IVS7-2A>G mutation (LQT1B) in *KCNQ1*. The baseline prolongations of field potential durations (FPDs) and action potential durations (APDs) were longer in LQT1-CMs than in WT-CMs. Exposure to low [K^+^]_Ex_ prolonged FPDs and APDs in a concentration-dependent fashion. LQT1-CMs were found to be more sensitive to low [K^+^]_Ex_ compared to WT-CMs. At baseline, LQT1A-CMs had more prolonged APDs than LQT1B-CMs, but low [K^+^]_Ex_ caused more pronounced APD prolongation in LQT1B-CMs. Early afterdepolarizations in the action potentials were observed in a subset of LQT1A-CMs with further prolonged baseline APDs and triangular phase 2 profiles. This work demonstrates that the hiPSC-derived CMs are sensitive to low [K^+^]_Ex_ and provide a platform to study acquired LQTS.

## Abstract (precis)

**Summary:** This is the first study showing the effects of low extracellular potassium on the electrophysiological properties of human induced pluripotent stem cell-derived long QT cardiomyocytes at single and multicellular level.

## INTRODUCTION

Long QT syndrome (LQTS) presents as an acquired or inherited arrhythmic disease characterized by prolonged QT interval on electrocardiogram and is associated with the occurrence of syncope or cardiac arrest. A special type of ventricular tachycardia known as torsades de pointes may arise in LQTS, which may degenerate into life-threatening ventricular fibrillation and cause sudden cardiac death (Schwartz et al., 2013).

Congenital forms of LQTS typically result from mutations in the cardiac ion channel-encoding genes. LQTS can be divided into different subtypes with LQT1 being the most common subtype caused by mutations in the *KCNQ1*. The *KCNQ1* encodes the *α*-subunit of the voltage-gated potassium channel and, assembled with auxiliary *β*-subunits encoded by *KCNE1*, conducts the slow delayed rectifier outward K^+^ current (I_Ks_) (Barhanin et al., 1996; Sanguinetti et al., 1996). The high prevalence (0.4%) of LQTS in Finland has been explained by four founder mutations in the Finnish population (Marjamaa et al., 2009). The most prevalent LQT1 causing founder mutations is the C-terminal *KCNQ1* G589D missense mutation (Piippo et al., 2001). Evidence suggests that G589D is a moderate dominant-negative trafficking mutation with normal functioning but with hindered transport to the cell membrane (Aromolaran et al., 2014). Another prevalent founder mutation is a strong dominant-negative splice site mutation IVS7-2A>G (Fodstad et al., 2006). Both, the G589D and IVS7-2A>G mutation types result in reduced I_Ks_ current when co-expressed independently with wild-type *KCNQ1* and *KCNE1* (Piippo et al., 2001; Fodstad et al., 2006).

Hypokalemia is one of the most common electrolyte disturbances and is characterized by low blood serum K^+^ levels. In normokalemic conditions, K^+^ concentrations range from 3.5-5.3 mM (Macdonald and Struthers, 2004). In moderate hypokalemia, the K^+^ concentrations range from 2.5-3.0 mM; and in severe hypokalemia they are <2.5 mM (Unwin et al., 2011). Hypokalemia is known to delay ventricular repolarization, slow ventricular conduction, cause hyperpolarization of the resting potential in ventricular myocytes as well as cause abnormal ventricular automaticity increasing the risk of ventricular arrhythmia and sudden cardiac death (Macdonald and Struthers, 2004; Osadchii, 2010; Schulman and Narins, 1990).

About 40% of the patients on thiazide diuretics have been reported to suffer from hypokalemia (Gennari, 1998) and a tenfold increase in hospital mortality was found in hypokalemic patients (Paltiel et al., 2001). Potassium electrolyte disturbance in serum is recognized as a risk factor among LQTS patients. The mortality rate of hospitalized hypokalemic patients was tenfold higher than in generalized hospital population illustrating the potentially life-threatening consequences of hypokalemia (Paltiel et al., 2001). Other studies on hypokalemia have reported prolonged QT-intervals, torsades de pointes and ventricular arrhythmias in LQT1 patients (Schulman and Narins, 1990; Roden, 1997). Under hypokalemic conditions, LQTS patients may be more susceptible to exaggerated QT-interval prolongation than healthy subjects because the repolarization reserve in LQTS patients is already compromised due to mutation(s) in the cardiac ion channel genes (Roden, 2004; Varró and Baczkó, 2011).

It is now possible to generate pluripotent stem cells (human induced pluripotent stem cells; hiPSCs) from any individual (Takahashi et al., 2007; Yu et al., 2007). hiPSCs have been shown to be a feasible research tool for the study of inherited cardiac diseases (e.g. LQTS) (Bellin et al., 2013; Moretti et al., 2010; Itzhaki et al., 2011; Lahti et al., 2012; Egashira et al., 2012; Matsa et al., 2011; Spencer et al., 2014; Malan et al., 2011; Yazawa et al., 2011; Ma et al., 2013; Kiviaho et al., 2015). The effects of low extracellular K^+^ concentrations ([K^+^]_Ex_) have currently not been investigated in hiPSC-derived LQT cardiomyocytes (CMs).

In this study, we have utilized a hiPSC-based model (Kiviaho et al., 2015) to study the effects of low [K^+^]_Ex_ in control CMs a well as in LQT1-specific CMs carrying Finnish founder mutations G589D or IVS7-2A>G. The electrophysiological properties of control subject (WT), and LQT1A (carrying G589D), LQT1B (carrying IVS7-2A>G) lines were assessed by determining the field potential durations (FPDs) and action potential durations (APDs), which correspond to surrogates for the QT-interval of the heart. The prolongation of APDs, FPDs and early afterdepolarizations (EADs) were assessed with low [K^+^]_Ex_ using ventricular-like CMs from WT, LQT1A and LQT1B lines.

## RESULTS

### Effect of [K^+^]_Ex_ on beating cardiomyocyte clusters

**Fig. 1.**
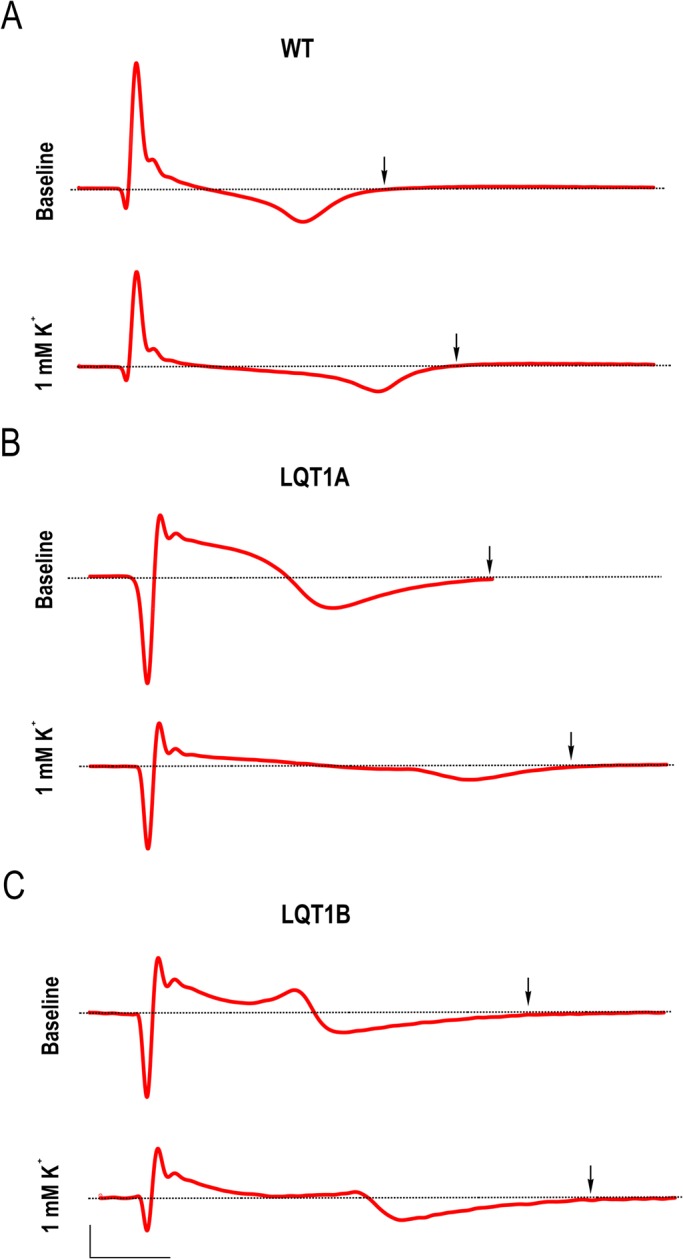
**Representative traces of field potentials at baseline and at 1 mM [K^+^]_Ex_ in cardiomyocyte clusters and the dose-dependent prolongation in field potentials corresponding to [K^+^]_Ex_.** (A) Recording of a representative field potential event by MEA at WT baseline (upper trace) and its corresponding field potential event in the presence of 1 mM [K^+^]_Ex_ (lower trace). (B) and (C) are equivalent to (A), but show LQT1A (B) and LQT1B (C), respectively. Scale bars: 40 µV (A), 10 µV (B) 10 µV in abscissa, (C) and the ordinate is 100 ms. Dashed lines (A-C) indicate 0 µV. Arrows indicate the endpoint of FPD.

Baseline electrophysiological properties of healthy control (WT) and patient-specific LQT1A and LQT1B CM clusters were assessed by multielectrode array (MEA) measurements at 5.33 mM extracellular K^+^ ([K^+^]_Ex_). Representative field potential traces from baseline (upper panels) of WT, LQT1A and LQT1B, respectively are depicted in Fig. 1A-C. Table 1A-C shows the FPD and corrected field potential durations (cFPDs) using two different correction formulae; Bazett and Fridericia. The Fridericia’s correction formula was used to describe the extracted MEA data and a comparison of baseline properties revealed that cFPDs of LQT1A and LQT1B were more prolonged compared to WT (*P*=1.8E-4, one-way ANOVA) and in the same test no difference in cFPDs between LQT1A and LQT1B were found (*P*=0.839, one-way ANOVA).

**Table 1.**
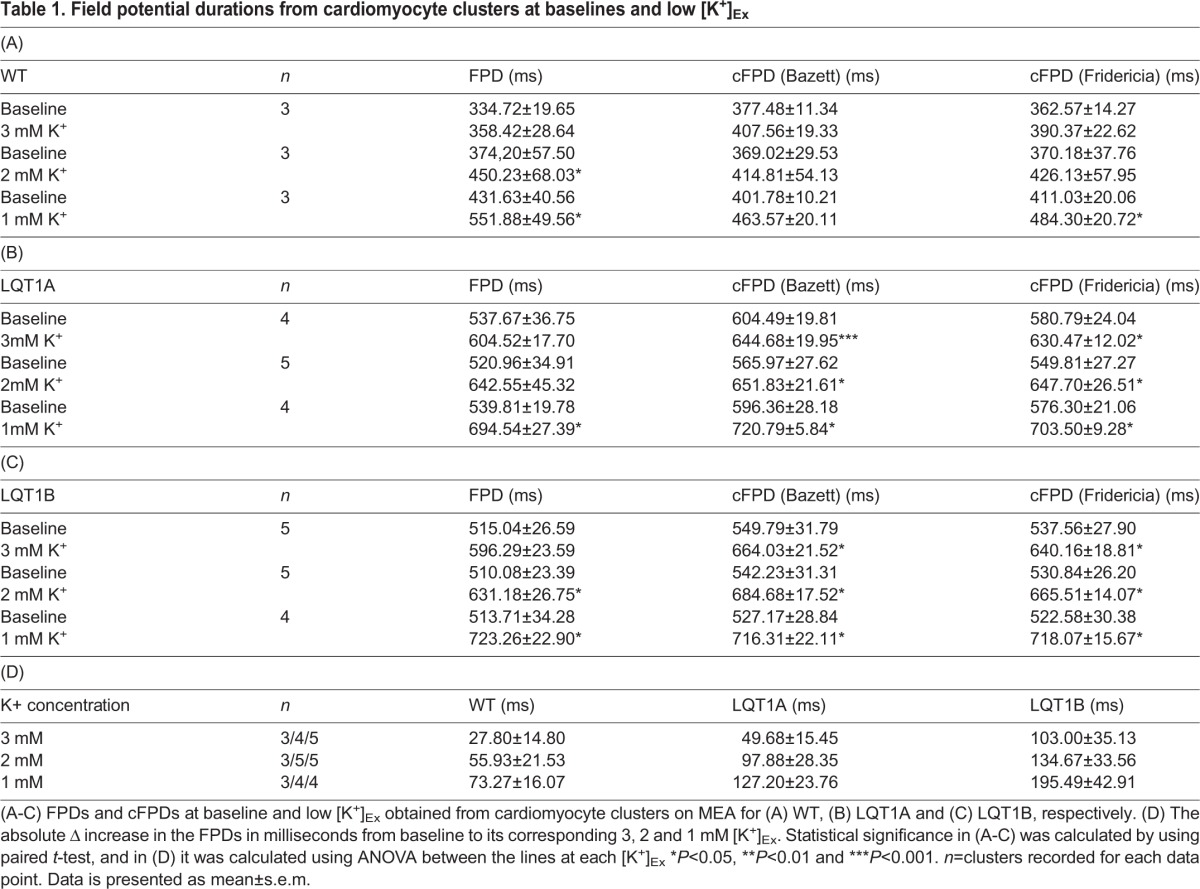
**Field potential durations from cardiomyocyte clusters at baselines and low [K^+^]_Ex_**

Following the investigation of baseline properties on the CM clusters, the effect of sequential reduction to 3, 2 and 1 mM [K^+^]_Ex_ were studied. Representative field potential traces from 1 mM [K^+^]_Ex_ (lower panels) of WT, LQT1A and LQT1B, respectively are depicted in Fig. 1A-C. A concentration-dependent increase in cFPD correlated with sequential reduction in [K^+^]_Ex_ (Table 1A-C). Table 1A shows that low [K^+^]_Ex_ prolonged cFPD in WT with significant difference (*P*=0.045, paired *t*-test) at 1 mM [K^+^]_Ex_.

In contrast, the cFPD was significantly prolonged in LQT1A (*P*=0.049, paired *t*-test) and LQT1B lines (*P*=0.043, paired *t*-test) already at 3 mM [K^+^]_ex_ (Table 1B,C). The absolute cFPD prolongation in response to lowered [K^+^]_Ex_ was calculated (Table 1D) but no statistically significant differences were found between the lines.

### Effect of [K^+^]_Ex_ on single beating cardiomyocytes

Similar to MEA, the electrophysiological properties of single CMs were compared among WT, LQT1A and LQT1B in current clamp. Table 2A-C shows extracted data from action potentials (AP) consisting of: action potential duration (APD), action potential amplitude (APA) and maximum diastolic potential (MDP). APD_90/50_ ratios were <1.30 indicating ventricular-like CM. The baseline APDs of LQT1A and LQT1B were significantly longer than WT (*P*<0.008, one-way ANOVA) and in the same test the APDs showed that LQT1A was significantly longer than LQT1B (*P*=0.007, *n*=22 in each group, one-way ANOVA). The baseline APD_50/10_ ratio was higher in LQT1A compared to WT and LQT1B, which indicates that phase 2 of the APs in LQT1A were significantly more triangular in shape (ratios: LQT1A 2.95±0.13**, *P*<0.002: LQT1B 2.45±0.09 and WT 2.28±0.07, *P*=0.438, one-way ANOVA, *n*=22, Table 2A-C).

**Table 2.**
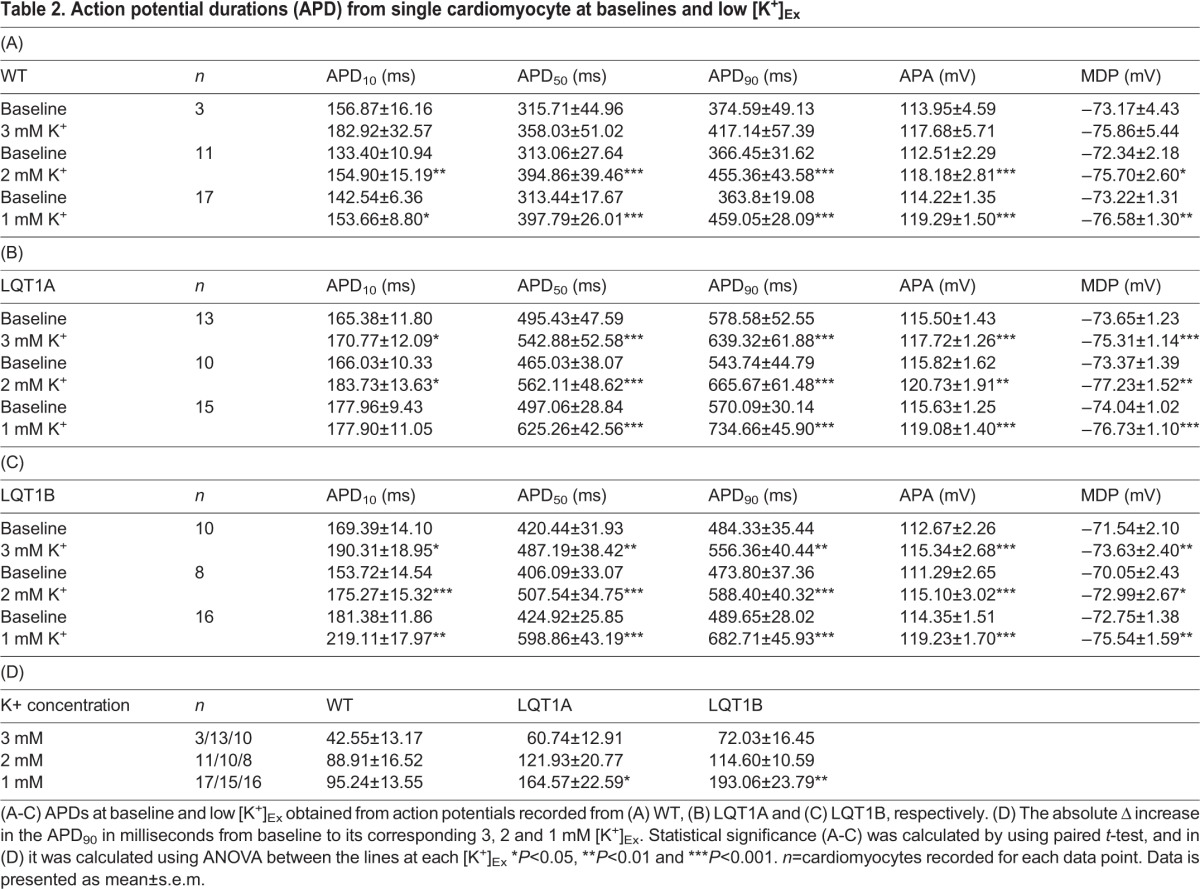
**Action potential durations (APD) from single cardiomyocyte at baselines and low [K^+^]_Ex_**

**Fig. 2.**
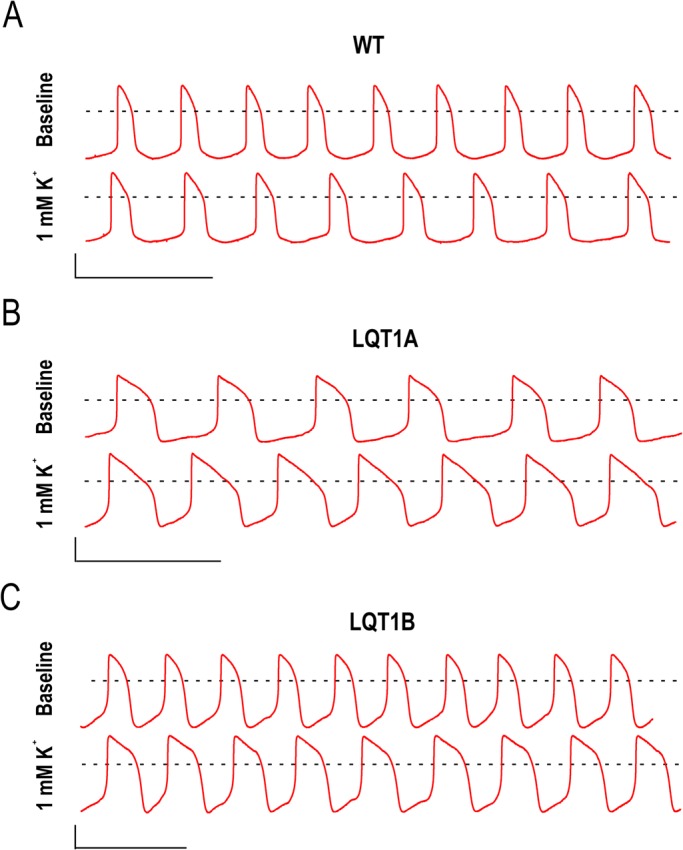
**Representative current clamp recordings of spontaneous actions potentials extracted from single ventricular-like cardiomyocyte baselines and their corresponding responses to 1 mM [K^+^]_Ex_ and dose-dependent prolongation of APD_90_ induced by [K^+^]_Ex_.** (A-C) Representative baselines (upper traces) and the corresponding effect of 1 mM [K^+^]_Ex_ (lower traces) for (A) WT, (B) LQT1A and (C) LQT1B, respectively. Scale bars: abscissa 40 mV and ordinate 2 s. Dashed lines (A-C) indicate 0 mV.

The [K^+^]_Ex_ in the perfusate was then lowered from 4.8 mM at baseline to 3, 2 or 1 mM and the effects were studied on the WT, LQT1A and LQT1B CMs. The APDs and APAs were increased and MDPs decreased at low [K^+^]_Ex_ (Table 2A-C). Fig. 2A-C show representative traces of baseline (upper trace) and 1 mM [K^+^]_Ex_ (lower trace). The relative prolongation of APD_90_ with low [K^+^]_Ex_ is shown in Fig. 2D and the APD_90_ of LQT1B CMs were found to be more prolonged compared to the LQT1A and WT CMs at 1 mM [K^+^]_Ex_ (*P*=0.025, one-way ANOVA). The absolute APD_90_ prolongation in response to lowered [K^+^]_Ex_ was calculated in the same data sets and is presented in Table 2D. This data reveals a more pronounced absolute APD_90_ prolongations with 1 mM in LQT1A and LQT1B lines compared to WT. This suggests that LQT1A is more prolonged in absolute terms while LQT1B is more sensitive to low [K^+^]_Ex_ in relative terms.

### Single LQT1A cardiomyocytes exhibit arrhythmia

**Fig. 3.**
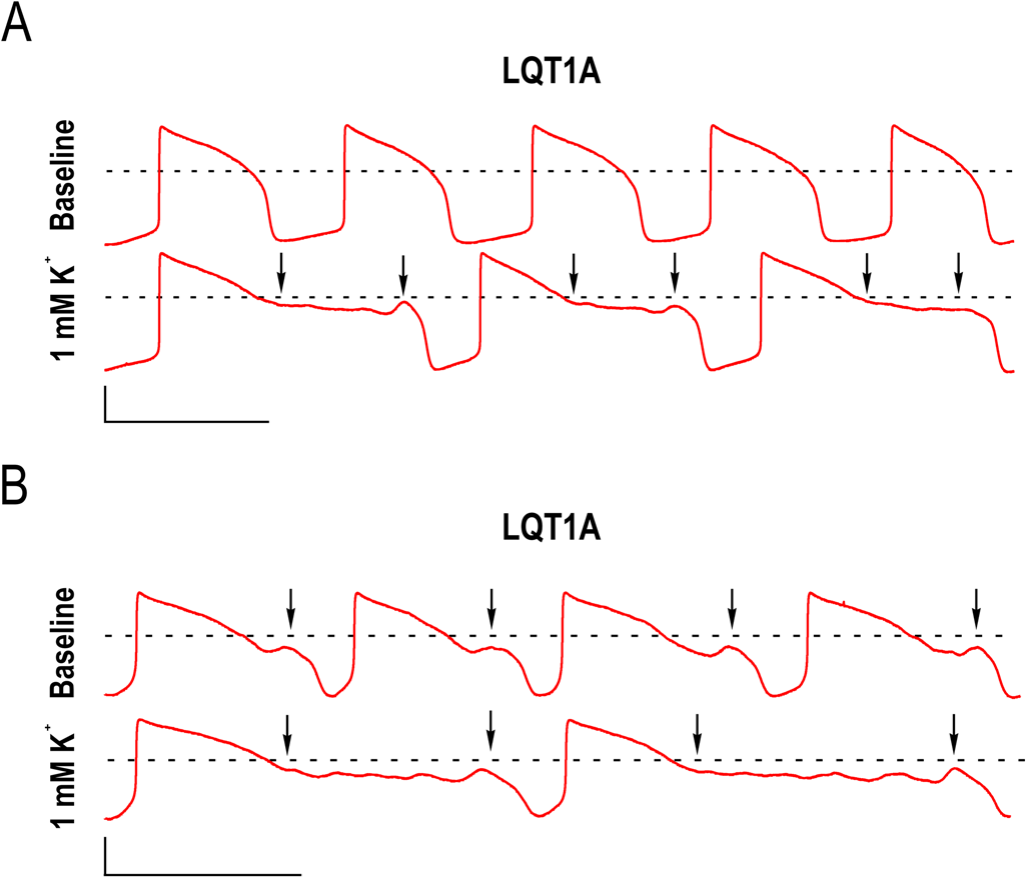
**Representative current clamp recordings of arrhythmia in LQT1A cardiomyocytes.** (A) A cardiomyocyte without early afterdepolarization (EAD) in baseline (upper trace) and with (EAD) only observed at 1 mM [K^+^]_Ex_ (lower trace). (B) Depicts a cardiomyocyte with EADs in baseline (upper trace) and EADs were prolonged at 1 mM [K^+^]_Ex_ (lower trace). Arrows point to the beginning and end of the arrhythmic Phase 2. Scale bars: 40 mV in abscissa and 2 s in ordinate. Dashed lines (A and B) indicate 0 mV. Note that EADs were only found in LQT1A cardiomyocytes with more prolonged baseline APDs (see Fig. 2B for a comparison).

Low [K^+^]_Ex_ did not evoke arrhythmias in WT, LQT1A and LQT1B CM clusters in MEA recordings. Similarly, in current clamp recordings no arrhythmias were found in WT and LQT1B. However, LQT1A exhibited characteristics shown in Fig. 3A,B. The APDs were further prolonged and exhibited early afterdepolarizations (EADs) evoked only with 1 mM [K^+^]_Ex_ (Table 3A). In some LQT1As, EADs were already present in baseline conditions (Table 3B). The total occurrence of LQT1A CMs exhibiting EADs was 37.5%. In baselines without EADs (but with EADs at low [K^+^]_Ex_), the APD_90/50_ were similar (<1.30) to CMs without EADs at low [K^+^]_Ex_. However, in the former (EADs at low [K^+^]_Ex_) the baseline APD_50/10_ ratios were higher than in the latter (CMs without the EADs) (*P*=1.3E-6, unpaired *t*-test, APD_50/10_ ratio: 4.83±0.68 Table 3A and 2.95±0.13 Table 2B, see also Figs 2B and 3A). Note, the CMs analyzed in Table 3A exhibited EADs only with 1 mM [K^+^]_Ex_. CMs with EADs occurring at baseline had an increase in EADs and APD prolongation with lower [K^+^]_Ex_ (Table 3B, Fig. 3B, arrows). The EAD ‘take off’ in baselines or with 1 mM [K^+^]_Ex_ occurred solely in phase 2 and were found to range from −15 mV to −27 mV. EAD ‘take off’ in baselines were −17.8±1.7 mV (*n*=5) and when evoked with 1 mM [K^+^]_Ex_ −20.3±1.3 mV (*n*=7), suggesting a role of L-type calcium current due to the fact that the triangular phase 2 is driven towards more negative membrane potential during further APD prolongation in these LQT1A CMs.

**Table 3.**
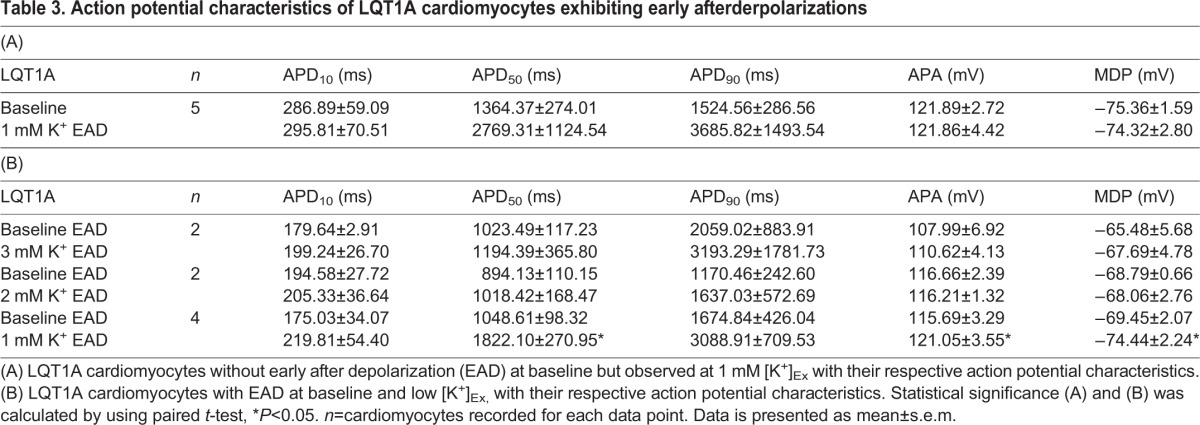
**Action potential characteristics of LQT1A cardiomyocytes exhibiting early afterderpolarizations**

## DISCUSSION

This study demonstrates the effect of low [K^+^]_Ex_ on WT, LQT1A and LQT1B CMs using MEA and current clamp. The baseline APD and FPD of LQT1A and LQT1B CMs were longer compared to WT. Prolonged repolarization was evoked in a concentration-dependent fashion by low [K^+^]_Ex_ in all types of CMs with the LQT1B CMs being most sensitive to low [K^+^]_Ex_. In current clamp we found that MDP was decreased (hyperpolarized) and APA was higher with low [K^+^]_Ex_. EADs were occasionally observed in LQT1A at single cell level and these CMs had further prolonged APDs already at baseline compared to the majority of LQT1A CMs. An interesting finding was that the phase 2 triangularity (APD_50/10_ ratio) was more pronounced in the LQT1A CMs at baseline when EADs were present. In support of our data, previous current clamp characteristics recorded at physiological potassium conditions at baseline are similar to WT, LQT1A and LQT1B CMs described in this study (Kiviaho et al., 2015). This is the first study where low potassium concentration induces prolonged repolarization in hiPSC-derived WT and LQTS CMs. Additionally, EADs were observed only in CMs with G589D point mutation (LQT1A), but not in those carrying the IVS7-2A>G mutations (LQT1B).

The appearance of EADs has been linked to the hyperpolarization of cell membrane in low [K^+^]_Ex_ conditions (Zaza, 2009). In the present study, we show that EADs similarly emerge from more hyperpolarized MDPs at low [K^+^]_Ex_. This is supported by an altered K^+^ conductance and electromotive force during acute and chronic low [K^+^]_Ex_ and is further strengthened by similar findings with data from animal ventricular cardiomyocytes (White and Terrar, 1991; Ruiz Ceretti et al., 1982; Kishida et al., 1979; Zaza, 2009), in transgenic rabbit LQT1 and LQT2 models (Liu et al., 2012) and in transgenic mouse LQT2 model (Teng et al., 2004). Our data with hiPSC-derived CMs thus correlates well with previous studies even though previously reported data were conducted with transgenic or non-human CMs.

In this study, low [K^+^]_Ex_ prolonged the FPD and APD in all our CMs (WT, LQT1A and LQT1B). This data is supported by previous findings with various animal studies as well as in isolated ventricular CMs (White and Terrar, 1991; Akita et al., 1998; Guo et al., 2011; Pezhouman et al., 2015; Chan et al., 2015; Osadchii, 2010). The prolongation of repolarization in hypokalemia (low [K^+^]_Ex_) has also been documented in cardiac patients receiving diuretic therapy (Stewart et al., 1985). It is well established that low [K^+^]_Ex_ suppresses the K^+^ currents such as I_Kr_ contributing to delayed repolarization thereby prolonging the QT-interval (Yang et al., 1997; Sanguinetti and Jurkiewicz, 1992; Scamps and Carmeliet, 1989; Guo et al., 2011; Osadchii, 2010). Thus, low extracellular K^+^ is recognized as a factor reducing the repolarization reserve (Varró and Baczkó, 2011).

The repolarization phase involves primarily I_Kr_ and I_Ks_ in ventricle myocytes. The LQT1 lines used in this study had mutation in the *KCNQ1* depressing the I_Ks_ current, which is presented as FPD and APD prolongations at baseline. Thus, this suggests prolongation in FPD and APD to increase in low [K^+^]_ex_ partly due to the lack of I_Ks_ repolarization reserve. The deficiency in I_Ks_ current in our LQT1 lines reproduces our previous findings where we show no or only a marginal effect on the APDs with a partial I_Ks_ blockade (JNJ303) in the LQT1 lines but robust effects in the WT line (Kiviaho et al., 2015). As expected, a partial I_Kr_ blockade (E4031) resulted in a prolongation of FPD and APD in the WT, LQT1A and LQT1B lines (Kiviaho et al., 2015; Pradhapan et al., 2013).

In a transgene expression system with the G589D point mutation, a reduced I_Ks_ current has been reported (Piippo et al., 2001). The G589D mutation has been found to be a trafficking defect (Aromolaran et al., 2014). The IVS7-2A>G splicing mutation is a ‘loss-of-function’ defect (Fodstad et al., 2006). Based on earlier expression studies in co-expression with wild-type *KCNQ1* and *KCNE1*, the IVS7-2A>G mutation decreases I_Ks_ current more than the G589D mutation (Piippo et al., 2001; Fodstad et al., 2006). Thus, these earlier findings support our data with difference in repolarization times, but do not explain the presence of EADs. This refers to complexity of K^+^ homeostasis and additional mechanisms in CMs.

The baseline FPD and APD in our data demonstrate that the repolarization time of LQT1A is longer than that of LQT1B, but LQT1B is more sensitive to low [K^+^]_Ex_ despite MDP and APA being similar in LQT1A and LQT1B CMs. Since I_Kr_ is a major player involved in repolarization, the simplest explanation would be that the I_Kr_ contribution to the repolarization reserve pool is larger in LQT1B than in LQT1A. The repolarization reserve pool of LQT1B would thus show greater K^+^ sensitivity due to suppressed K^+^ conductance, increased electromotive force when acutely exposed to low [K^+^]_Ex_ (Zaza, 2009) and no I_Ks_ functionality. This would also explain why LQT1B has shorter FPD and APD baseline values compared to LQT1A in this study. Moreover, this would be in line with our previous finding that EADs in LQT1B are more pronounced compared to LQT1A during an I_Kr_ blockade with E4031 at normal physiological K^+^ conditions (Kiviaho et al., 2015). As I_Ks_ is diminished in our LQT1 lines, the I_Kr_ functionality will become important for the repolarization reserve during development of hypokalemia. The acute and chronic K^+^ homeostasis is complex with effects in a variety of parameters. Based on our studies we can exclude I_Ks_ as a major contributor in the repolarization reserve in the LQT1 lines. This is supported by the fact that only LQT1A CMs present a prolonged phase 2 triangularity and EADs, but not LQT1B CMs which, however, have less I_Ks_ current based on gene transfection experiments (Piippo et al., 2001; Fodstad et al., 2006).

Action potential shape has been suggested to play a crucial role in occurrence of pro-arrhythmic events (Hondeghem et al., 2001; Osadchii and Olesen, 2009). Phase 3 triangularity is shown to be a marker for pro-arrhythmia in monophasic action potentials of guinea pig and rabbit hearts (Hondeghem et al., 2001; Osadchii and Olesen, 2009). I_Kr_ deficiency has also been associated in phase 3 triangularity (Hondeghem et al., 2001); however, in this study, we did not find EADs evoked in phase 3 but in phase 2 suggesting a reopening of L-type calcium channels (I_Ca,L_), i.e. in the LQT1A CMs with EADs, these repeatedly occurred between –15 mV and –27 mV. It has previously been suggested that phase 2 EADs are carried by I_Ca,L_ following an APD prolongation towards a more negative membrane potential while we cannot exclude that the Na^+^/Ca^2+^-exchanger works in concert with the I_Ca,L_ (Guo et al., 2007; Sipido et al., 2007; Banyasz et al., 2012). It is currently unknown what causes the further prolongation and triangularity in phase 2 giving rise to EADs found in our LQT1A CMs. It should be noted that no arrhythmia was detected and no phase 2 triangularity was observed in LQT1B and WT CMs.

### Potential limitations of the study

The effects of low [K^+^]_Ex_ on hiPSC-derived CM may not completely reflect the symptoms of hypokalemia *in vivo*. hiPSC-derived CMs are still more fetal-like and this has to be considered when translating these results to the clinics. I_Ks_ and I_Kr_ have not been quantified in our study but their functionality has been analyzed with the use of specific ion channel blockers in our previous study (Kiviaho et al., 2015). Further understanding the interaction between ion channels and exchangers is needed for revealing the underlying mechanisms of prolongation and arrhythmia observed in the LQT1 lines used in this study. Here, we only have an acute expose and long term exposure to low [K^+^]_Ex_ on hiPSC-derived CMs may reveal different characteristics.

In conclusion, we have shown that low [K^+^]_Ex_ delays the repolarization in hiPSC-derived CMs at single cell as well as at multicellular level. The LQT1 CMs are more sensitive to potassium electrolyte disturbances than WT CMs, thus confirming previous clinical studies. In LQT1 CMs with G589D mutation phase 2 triangularity in EADs were occasionally observed at baseline or could be further evoked by low [K^+^]_Ex_. However, phase 2 triangularity or EADs could not be evoked in LQT1 CMs with IVS7-2A>G mutation or in WT CMs. The effects of low [K^+^]_Ex_ has not previously been investigated in hiPSC-derived CMs. Thus, this work demonstrates that the hiPSC-derived CMs provide a platform for studying the effects of low extracellular K^+^ and also provide a platform to study acquired LQTS.

## MATERIAL AND METHODS

### Ethical approval

The study was approved by the Ethics Committee of Pirkanmaa Hospital District to establish, culture, and differentiate hiPSC lines (R08070). Participants donating skin biopsies signed an informed consent after receiving oral and written descriptions of the study. Skin biopsies for hiPSC establishment were received from the Heart Hospital, Tampere University Hospital, Tampere, Finland.

### Long QT patient characteristics

The LQT patient characteristics have been previously described elsewhere (Kiviaho et al., 2015). In brief, the LQT1 patient carrying G589D mutation is a 46-year-old female with corrected QT interval (QTc) of 464 ms. The other LQT1 patient carrying IVS7-2A>G is a 51-year-old female with QTc value of 489 ms. These patients are symptomatic with episodes of syncope.

### Human induced pluripotent stem cell generation and culture

Patient-specific hiPSCs were generated as described earlier (Takahashi et al., 2007). The LQT1-specific hiPSCs were derived from patients carrying G589D or IVS7-2A>G mutation in the *KCNQ1* as previously described (Kiviaho et al., 2015). Healthy control hiPSCs (WT) were derived from skin fibroblasts of a healthy 55-year-old female (Ahola et al., 2014). hiPSC lines were cultured in knockout serum replacement medium using mouse embryonic fibroblasts (Millipore, Billerica, MA, USA) as feeders. The following components were included in the knockout serum replacement medium: knockout-DMEM (Invitrogen, Carlsbad, CA, USA) containing 20% knockout-serum replacement (Invitrogen, Carlsbad, CA, USA), nonessential amino acids, GlutaMAX, penicillin/streptomycin, 0.1 mM 2-mercaptoethanol and 4 ng/ml fibroblast growth factor 2 (R&D Systems Inc., Minneapolis, MN, USA). The medium was refreshed three times a week and the hiPSCs were passaged weekly using collagenase IV (Invitrogen, Carlsbad, CA, USA).

### Cell line characterizations and cardiomyocyte differentiation

The hiPSC lines and hiPSC-derived CMs used in this study have been characterized elsewhere. The control cell line UTA.04602.WT (WT) and WT-derived CMs have been characterized in detail previously (Ahola et al., 2014). The hiPSC lines UTA.00208.LQT1 (LQT1A, derived from patient carrying G589D mutation) and UTA.00118.LQT1 (LQT1B, derived from patient carrying IVS7-2A>G mutation) and CMs derived from them have been characterized (Kiviaho et al., 2015). The hiPSCs were differentiated into CMs in co-culture with mouse visceral endoderm-like cells. The differentiation method has been described elsewhere (Mummery et al., 2003).

### Multielectrode array recordings and data analysis

hiPSC-derived CMs (30-35 days old) were used in this study. Spontaneously beating CM clusters were manually dissected and plated on 6-well MEAs (6-well MEA 200/30iR-Ti-tcr, Multichannel Systems, Reutlingen, Germany), which were first coated with fetal bovine serum (FBS, Invitrogen, Carlsbad, CA, USA) for 30 min at room temperature and then with 0.1% gelatin (Sigma Aldrich, St Louis, MO, USA) for 1 h at room temperature. The CM clusters were plated in EB-medium consisting of: knockout-DMEM with 20% FBS, nonessential amino acids, GlutaMAX and penicillin/streptomycin. Custom made K^+^ free DMEM (Irvine Scientific, CA, USA) supplemented with varying concentrations of K^+^ (B. Braun, Melsungen, Germany, KCl 150 mg/ml) and penicillin/streptomycin were used in this study. The following [K^+^]_Ex_ were used: 5.33, 4, 3, 2 and 1 mM. The EB-medium was changed to serum-free culture medium (5.33 mM K^+^) and after the incubation of 1 h, the field potentials originating from the CMs were recorded (baseline). After baseline recordings, the [K^+^]_Ex_ in the medium was sequentially reduced from 4 mM to 1 mM by medium exchanges with 45 min incubations in between each concentration and recorded at 37°C using MEAs (MEA1060-Inv-BC, Multichannel Systems, Reutlingen, Germany) using 20 kHz sampling frequency and MC_Rack software (Multichannel Systems, Reutlingen, Germany). Note, 4 mM induced no or only marginal effects on FPD (data not shown), thus it has not been included to the result section for clarity of the presentation. The MEAs were covered with gas permeable membranes (ALA MEA-Sheet, ALA Scientific, NY, USA) during the recordings. The total recording time for each concentration was 2 min. The MEA data was analyzed by in-house-developed CardioMDA software (Pradhapan et al., 2013). CardioMDA is available from BioMediTech website (http://www.biomeditech.fi/CardioMDA/). The Bazett's and Fridericia's formulae were used to calculate the beat rate corrected FPD. The criteria for data analysis of the MEA recordings was that only the aggregates responding to low [K^+^]_Ex_ with FPD prolongation were included in this study.

### Current clamp

Current clamp recordings were performed as previously described (Kiviaho et al., 2015). In brief, the perforated patch clamp technique was used with amphotericin-B as perforation agent with a final concentration of 240 µg/ml (Hamill et al., 1981; Rae et al., 1991). Action potentials were acquired with an Axon Series 200B amplifier and a Digidata 1440 AD/DA converter (Molecular device, LTD, USA). Dissociated CMs were plated on 5 mmØ coverslips and recorded on the 6th or 7th day. In brief, coverslips were transferred to a RC-24N recording chamber (Harvard Instruments UK, Warner Instruments, USA) and placed on an inverted Olympus IX71 microscope. The perfusate was maintained at 35-36°C using an SH-27B inline heater (Harvard Apparatus Ltd., Kent, UK). The HEPES based extracellular solution (HBS) contained (in mM): 143 NaCl, 1.8 CaCl_2_, 1.2 MgCl_2_, 5 glucose and 10 HEPES. For experiments KCl was added to the HBS to final concentrations of 1, 2, 3 and 4.8 mM K^+^ (pH was set to 7.4 with NaOH and osmolarity set to 300-302 mOsm with sucrose). HBS with 4.8 mM K^+^ was used in baseline recordings. The pipette solution contained (in mM): 122 KMeSO_4_, 30 KCl, 1.2 MgCl_2_, 1 CaCl_2_ (pH was set to 7.2 with KOH and osmolarity set to 290-292mOsm with sucrose). The pipette resistance was ∼3MΩ after filling with pipette solution. The action potentials from spontaneously beating CMs were recorded in current clamp mode. Current clamp recordings were digitally sampled at 20 kHz and filtered at 2 kHz using low pass Bessel filter on recording amplifier. AP duration at 10, 50 and 90% repolarization (APD_10_, APD_50_ and APD_90_), APA, and MDP were extracted from recorded action potentials using in house developed analysis modules running on the Origin 9 platform (Microcal Origin^TM^, Northampton, Massachusetts, USA). Baseline data in a CM was extracted from a minimum of 15 single APs and summed for statistics. When EADs were present in a CM, a minimum of 5 single APs were obtained in regions with maximal effect of [K^+^]_Ex_ and summed for each concentration for statistics. Acute application of [K^+^]_Ex_ was tested in sequential application without wash out, with wash out and with single applications from baselines to either 3, 2 or 1 mM [K^+^]_Ex_. Maximal effect was observed in seconds and recordings were fully reversible, thus data is pools of these series. The data presented from current clamp was from ventricular-like CMs. These were grouped as ventricular-like when baseline APs had APD_90/50_ <1.3, APA >100 mV and MDP <−60 mV. The Phase 2 AP triangularity was calculated as APD_50/10_.

### Statistical analyses

One-way ANOVA followed by Tukey’s post hoc test was used to assess differences in means of groups of three pairs. Paired *t*-test was used to access difference between the means in baseline and the corresponding effect of 3, 2 or 1 mM K^+^ in the same recording, while unpaired *t*-test was uses to access differences in means of two similar recordings (Microcal Origin™ 9.1, Northampton, Massachusetts, USA). Significant difference in the tables is presented as *P<0.05, **P<0.01 and ***P<0.001 and data is presented as mean±s.e.m.

## References

[BIO024216C1] Ahola A., Kiviaho A. L., Larsson K., Honkanen M., Aalto-Setälä K. and Hyttinen J. (2014). Video image-based analysis of single human induced pluripotent stem cell derived cardiomyocyte beating dynamics using digital image correlation. *Biomed. Eng. Online* 13, 39 10.1186/1475-925x-13-39 24708714 PMC3984432

[BIO024216C2] Akita M., Kuwahara M., Tsubone H. and Sugano S. (1998). ECG changes during furosemide-induced hypokalemia in the rat. *J. Electrocardiol.* 31, 45-49. 10.1016/S0022-0736(98)90006-1 9533377

[BIO024216C3] Aromolaran A. S., Subramanyam P., Chang D. D., Kobertz W. R. and Colecraft H. M. (2014). LQT1 mutations in KCNQ1 C-terminus assembly domain suppress IKs using different mechanisms. *Cardiovasc. Res.* 104, 501-511. 10.1093/cvr/cvu231 25344363 PMC4296111

[BIO024216C5] Banyasz T., Horvath B., Jian Z., Izu L. T. and Chen-Izu Y. (2012). Profile of L-type Ca(2+) current and Na(+)/Ca(2+) exchange current during cardiac action potential in ventricular myocytes. *Heart Rhythm.* 9, 134-142. 10.1016/j.hrthm.2011.08.029 21884673 PMC3252888

[BIO024216C6] Barhanin J., Lesage F., Guillemare E., Fink M., Lazdunski M. and Romey G. (1996). K(V)LQT1 and lsK (minK) proteins associate to form the I(Ks) cardiac potassium current. *Nature* 384, 78-80. 10.1038/384078a0 8900282

[BIO024216C7] Bellin M., Casini S., Davis R. P., D'Aniello C., Haas J., Ward-van Oostwaard D., Tertoolen L. G. J., Jung C. B., Elliott D. A., Welling A. et al. (2013). Isogenic human pluripotent stem cell pairs reveal the role of a KCNH2 mutation in long-QT syndrome. *EMBO J.* 32, 3161-3175. 10.1038/emboj.2013.240 24213244 PMC3981141

[BIO024216C8] Chan Y.-H., Tsai W.-C., Ko J.-S., Yin D., Chang P.-C., Rubart M., Weiss J. N., Everett T. H., Lin S.-F. and Chen P.-S. (2015). Small-conductance calcium-activated potassium current is activated during hypokalemia and masks short-term cardiac memory induced by ventricular pacing. *Circulation* 132, 1377-1386. 10.1161/CIRCULATIONAHA.114.015125 26362634 PMC4605871

[BIO024216C9] Egashira T., Yuasa S., Suzuki T., Aizawa Y., Yamakawa H., Matsuhashi T., Ohno Y., Tohyama S., Okata S., Seki T. et al. (2012). Disease characterization using LQTS-specific induced pluripotent stem cells. *Cardiovasc. Res.* 95, 419-429. 10.1093/cvr/cvs206 22739119

[BIO024216C10] Fodstad H., Bendahhou S., Rougier J. S., Laitinen-Forsblom P. J., Barhanin J., Abriel H., Schild L., Kontula K. and Swan H. (2006). Molecular characterization of two founder mutations causing long QT syndrome and identification of compound heterozygous patients. *Ann. Med.* 38, 294-304. 10.1080/07853890600756065 16754261

[BIO024216C11] Gennari F. J. (1998). Hypokalemia. *New Engl. J. Med.* 339, 451-458. 10.1056/NEJM199808133390707 9700180

[BIO024216C12] Guo D., Zhao X., Wu Y., Liu T., Kowey P. R. and Yan G.-X. (2007). L-type calcium current reactivation contributes to arrhythmogenesis associated with action potential triangulation. *J. Cardiovasc. Electrophysiol.* 18, 196-203. 10.1111/j.1540-8167.2006.00698.x 17212595

[BIO024216C14] Guo J., Wang T., Yang T., Xu J., Li W., Fridman M. D., Fisher J. T. and Zhang S. (2011). Interaction between the cardiac rapidly (I Kr) and slowly (I Ks) activating delayed rectifier potassium channels revealed by low K +-induced herg endocytic degradation. *J. Biol. Chem.* 286, 34664-34674. 10.1074/jbc.M111.253351 21844197 PMC3186392

[BIO024216C15] Hamill O. P., Marty A., Neher E., Sakmann B. and Sigworth F. J. (1981). Improved patch-clamp techniques for high-resolution current recording from cells and cell-free membrane patches. *Pflugers Arch.* 391, 85-100. 10.1007/BF00656997 6270629

[BIO024216C16] Hondeghem L. M., Carlsson L. and Duker G. (2001). Instability and triangulation of the action potential predict serious proarrhythmia, but action potential duration prolongation is antiarrhythmic. *Circulation* 103, 2004-2013. 10.1161/01.CIR.103.15.2004 11306531

[BIO024216C17] Itzhaki I., Maizels L., Huber I., Zwi-Dantsis L., Caspi O., Winterstern A., Feldman O., Gepstein A., Arbel G., Hammerman H. et al. (2011). Modelling the long QT syndrome with induced pluripotent stem cells. *Nature* 471, 225-229. 10.1038/nature09747 21240260

[BIO024216C18] Kishida H., Surawicz B. and Fu L. T. (1979). Effects of K+ and K+-induced polarization on (dV/dt)max, threshold potential, and membrane input resistance in guinea pig and cat ventricular myocardium. *Circ. Res.* 44, 800-814. 10.1161/01.RES.44.6.800 428073

[BIO024216C19] Kiviaho A. L., Ahola A., Larsson K., Penttinen K., Swan H., Pekkanen-Mattila M., Venäläinen H., Paavola K., Hyttinen J. and Aalto-Setälä K. (2015). Distinct electrophysiological and mechanical beating phenotypes of long QT syndrome type 1-specific cardiomyocytes carrying different mutations. *Int. J. Cardiol. Heart Vasc.* 8, 19-31. 10.1016/j.ijcha.2015.04.008 PMC5497295 28785673

[BIO024216C20] Lahti A. L., Kujala V. J., Chapman H., Koivisto A.-P., Pekkanen-Mattila M., Kerkela E., Hyttinen J., Kontula K., Swan H., Conklin B. R. et al. (2012). Model for long QT syndrome type 2 using human iPS cells demonstrates arrhythmogenic characteristics in cell culture. *Dis. Model. Mech.* 5, 220-230. 10.1242/dmm.008409 22052944 PMC3291643

[BIO024216C21] Liu G.-X., Choi B.-R., Ziv O., Li W., de Lange E., Qu Z. and Koren G. (2012). Differential conditions for early after-depolarizations and triggered activity in cardiomyocytes derived from transgenic LQT1 and LQT2 rabbits. *J. Physiol.* 590, 1171-1180. 10.1113/jphysiol.2011.218164 22183728 PMC3381823

[BIO024216C22] Ma D., Wei H., Zhao Y., Lu J., Li G., Sahib N. B. E., Tan T. H., Wong K. Y., Shim W., Wong P. et al. (2013). Modeling type 3 long QT syndrome with cardiomyocytes derived from patient-specific induced pluripotent stem cells. *Int. J. Cardiol.* 168, 5277-5286. 10.1016/j.ijcard.2013.08.015 23998552

[BIO024216C23] Macdonald J. E. and Struthers A. D. (2004). What is the optimal serum potassium level in cardiovascular patients? *J. Am. Coll. Cardiol.* 43, 155-161. 10.1016/j.jacc.2003.06.021 14736430

[BIO024216C24] Malan D., Friedrichs S., Fleischmann B. K. and Sasse P. (2011). Cardiomyocytes obtained from induced pluripotent stem cells with Long-QT syndrome 3 recapitulate typical disease-specific features in vitro. *Circ. Res.* 109, 841-847. 10.1161/CIRCRESAHA.111.243139 21799153

[BIO024216C25] Marjamaa A., Salomaa V., Newton-Cheh C., Porthan K., Reunanen A., Karanko H., Jula A., Lahermo P., Väänänen H., Toivonen L. et al. (2009). High prevalence of four long QT syndrome founder mutations in the finnish population. *Ann. Med.* 41, 234-240. 10.1080/07853890802668530 19160088 PMC2704397

[BIO024216C26] Matsa E., Rajamohan D., Dick E., Young L., Mellor I., Staniforth A. and Denning C. (2011). Drug evaluation in cardiomyocytes derived from human induced pluripotent stem cells carrying a long QT syndrome type 2 mutation. *Eur. Heart J.* 32, 952-962. 10.1093/eurheartj/ehr073 21367833 PMC3076668

[BIO024216C27] Moretti A., Bellin M., Welling A., Jung C. B., Lam J. T., Bott-Flügel L., Dorn T., Goedel A., Höhnke C., Hofmann F. et al. (2010). Patient-specific induced pluripotent stem-cell models for long-QT syndrome. *New Engl. J. Med.* 363, 1397-1409. 10.1056/NEJMoa0908679 20660394

[BIO024216C28] Mummery C., Ward-van Oostwaard D., Doevendans P., Spijker R., van den Brink S., Hassink R., van der Heyden M., Opthof T., Pera M., de la Riviere A. B. et al. (2003). Differentiation of human embryonic stem cells to cardiomyocytes: role of coculture with visceral endoderm-like cells. *Circulation* 107, 2733-2740. 10.1161/01.CIR.0000068356.38592.68 12742992

[BIO024216C29] Osadchii O. E. (2010). Mechanisms of hypokalemia-induced ventricular arrhythmogenicity. *Fundam. Clin. Pharmacol.* 24, 547-559. 10.1111/j.1472-8206.2010.00835.x 20584206

[BIO024216C30] Osadchii O. E. and Olesen S. P. (2009). Electrophysiological determinants of hypokalaemia-induced arrhythmogenicity in the guinea-pig heart. *Acta Physiol.* 197, 273-287. 10.1111/j.1748-1716.2009.02028.x 19656123

[BIO024216C31] Paltiel O., Salakhov E., Ronen I., Berg D. and Israeli A. (2001). Management of severe hypokalemia in hospitalized patients: a study of quality of care based on computerized databases. *Arch. Intern. Med.* 161, 1089-1095. 10.1001/archinte.161.8.1089 11322843

[BIO024216C32] Pezhouman A., Singh N., Song Z., Nivala M., Eskandari A., Cao H., Bapat A., Ko C. Y., Nguyen T. P., Qu Z. et al. (2015). Molecular basis of hypokalemia-induced ventricular fibrillation. *Circulation* 132, 1528-1537. 10.1161/CIRCULATIONAHA.115.016217 26269574 PMC4618042

[BIO024216C33] Piippo K., Swan H., Pasternack M., Chapman H., Paavonen K., Viitasalo M., Toivonen L. and Kontula K. (2001). A founder mutation of the potassium channel KCNQ1 in long QT syndrome: Implications for estimation of disease prevalence and molecular diagnostics. *J. Am. Coll. Cardiol.* 37, 562-568. 10.1016/S0735-1097(00)01124-4 11216980

[BIO024216C34] Pradhapan P., Kuusela J., Viik J., Aalto-Setälä K. and Hyttinen J. (2013). Cardiomyocyte MEA data analysis (CardioMDA)--a novel field potential data analysis software for pluripotent stem cell derived cardiomyocytes. *PLoS One* 8, e73637 10.1371/journal.pone.0073637 24069215 PMC3777951

[BIO024216C35] Rae J., Cooper K., Gates P. and Watsky M. (1991). Low access resistance perforated patch recordings using amphotericin B. *J. Neurosci. Methods.* 37, 15-26. 10.1016/0165-0270(91)90017-T 2072734

[BIO024216C36] Roden D. M. (1997). A practical approach to torsade de pointes. *Clin. Cardiol.* 20, 285-290. 10.1002/clc.4960200318 9068917 PMC6656095

[BIO024216C37] Roden D. M. (2004). Drug-induced prolongation of the QT interval. *New Engl. J. Med.* 350, 1013-1022. 10.1056/NEJMra032426 14999113

[BIO024216C38] Ruiz Ceretti E., Ponce Zumino A., Blaney R. and Chartier D. (1982). Experimental arrhythmia elicited by low K perfusion. *Can. J. Physiol. Pharmacol.* 60, 1533-1540. 10.1139/y82-226 7165850

[BIO024216C39] Sanguinetti M. C. and Jurkiewicz N. K. (1992). Role of external Ca2+ and K+ in gating of cardiac delayed rectifier K+ currents. *Pflügers Arch.* 420, 180-186. 10.1007/BF00374988 1620577

[BIO024216C40] Sanguinetti M. C., Curran M. E., Zou A., Shen J., Spector P. S., Atkinson D. L. and Keating M. T. (1996). Coassembly of K(V)LQT1 and minK (IsK) proteins to form cardiac I(Ks) potassium channel. *Nature* 384, 80-83. 10.1038/384080a0 8900283

[BIO024216C41] Scamps F. and Carmeliet E. (1989). Delayed K+ current and external K+ in single cardiac Purkinje cells. *Am. J. Physiol. Cell Physiol.* 257, C1086-C1082. 10.1152/ajpcell.1989.257.6.C1086 2610249

[BIO024216C42] Schulman M. and Narins R. G. (1990). Hypokalemia and cardiovascular disease. *Am. J. Cardiol.* 65 10.1016/0002-9149(90)90244-u 2178377

[BIO024216C43] Schwartz P. J., Ackerman M. J., George A. L. Jr and Wilde A. A. M. (2013). Impact of genetics on the clinical management of channelopathies. *J. Am. Coll. Cardiol.* 62, 169-180. 10.1016/j.jacc.2013.04.044 23684683 PMC3710520

[BIO024216C44] Sipido K. R., Bito V., Antoons G., Volders P. G. and Vos M. A. (2007). Na/Ca exchange and cardiac ventricular arrhythmias. *Ann. New York Acad. Sci.* 1099, 339-348. 10.1196/annals.1387.066 17446474

[BIO024216C45] Spencer C. I., Baba S., Nakamura K., Hua E. A., Sears M. A. F., Fu C.-C., Zhang J., Balijepalli S., Tomoda K., Hayashi Y. et al. (2014). Calcium transients closely reflect prolonged action potentials in iPSC models of inherited cardiac arrhythmia. *Stem Cell Rep.* 3, 269-281. 10.1016/j.stemcr.2014.06.003 PMC4175159 25254341

[BIO024216C46] Stewart D. E., Ikram H., Espiner E. A. and Nicholls M. G. (1985). Arrhythmogenic potential of diuretic induced hypokalaemia in patients with mild hypertension and ischaemic heart disease. *Br. Heart J.* 54, 290-297. 10.1136/hrt.54.3.290 4041299 PMC481898

[BIO024216C47] Takahashi K., Tanabe K., Ohnuki M., Narita M., Ichisaka T., Tomoda K. and Yamanaka S. (2007). Induction of pluripotent stem cells from adult human fibroblasts by defined factors. *Cell* 131, 861-872. 10.1016/j.cell.2007.11.019 18035408

[BIO024216C48] Teng G., Zhao X., Cross J. C., Li P., Lees-Miller J. P., Guo J., Dyck J. R. B. and Duff H. J. (2004). Prolonged repolarization and triggered activity induced by adenoviral expression of HERG N629D in cardiomyocytes derived from stem cells. *Cardiovasc. Res.* 61, 268-277. 10.1016/j.cardiores.2003.11.016 14736543

[BIO024216C49] Unwin R. J., Luft F. C. and Shirley D. G. (2011). Pathophysiology and management of hypokalemia: a clinical perspective. *Nat. Rev. Nephrol.* 7, 75-84. 10.1038/nrneph.2010.175 21278718

[BIO024216C50] Varró A. and Baczkó I. (2011). Cardiac ventricular repolarization reserve: a principle for understanding drug-related proarrhythmic risk. *Br. J. Pharmacol.* 164, 14-36. 10.1111/j.1476-5381.2011.01367.x 21545574 PMC3171857

[BIO024216C51] White E. and Terrar D. A. (1991). Action potential duration and the inotropic response to reduced extracellular potassium in guinea-pig ventricular myocytes. *Exp. Physiol.* 76, 705-716. 10.1113/expphysiol.1991.sp003537 1742012

[BIO024216C52] Yang T., Snyders D. J. and Roden D. M. (1997). Rapid inactivation determines the rectification and [K+](o) dependence of the rapid component of the delayed rectifier K+ current in cardiac cells. *Circ. Res.* 80, 782-789. 10.1161/01.RES.80.6.782 9168780

[BIO024216C53] Yazawa M., Hsueh B., Jia X., Pasca A. M., Bernstein J. A., Hallmayer J. and Dolmetsch R. E. (2011). Using induced pluripotent stem cells to investigate cardiac phenotypes in Timothy syndrome. *Nature* 471, 230-234. 10.1038/nature09855 21307850 PMC3077925

[BIO024216C54] Yu J., Vodyanik M. A., Smuga-Otto K., Antosiewicz-Bourget J., Frane J. L., Tian S., Nie J., Jonsdottir G. A., Ruotti V., Stewart R. et al. (2007). Induced pluripotent stem cell lines derived from human somatic cells. *Science* 318, 1917-1920. 10.1126/science.1151526 18029452

[BIO024216C55] Zaza A. (2009). Serum potassium and arrhythmias. *Europace* 11, 421-422. 10.1093/europace/eup005 19182234

